# Optimized microfluidic formulation and organic excipients for improved lipid nanoparticle mediated genome editing

**DOI:** 10.1039/d4lc00283k

**Published:** 2024-07-22

**Authors:** Rohan Palanki, Emily L. Han, Amanda M. Murray, Rohin Maganti, Sophia Tang, Kelsey L. Swingle, Dongyoon Kim, Hannah Yamagata, Hannah C. Safford, Kaitlin Mrksich, William H. Peranteau, Michael J. Mitchell

**Affiliations:** a Department of Bioengineering, University of Pennsylvania Philadelphia PA 19104 USA mjmitch@seas.upenn.edu; b Center for Fetal Research, Children's Hospital of Philadelphia Philadelphia PA 19104 USA peranteauw@chop.edu; c Abramson Cancer Center, Perelman School of Medicine, University of Pennsylvania Philadelphia PA 19104 USA; d Center for Cellular Immunotherapies, Perelman School of Medicine, University of Pennsylvania Philadelphia PA 19104 USA; e Penn Institute for RNA Innovation, Perelman School of Medicine, University of Pennsylvania Philadelphia PA 19104 USA; f Institute for Immunology, Perelman School of Medicine, University of Pennsylvania Philadelphia PA 19104 USA; g Cardiovascular Institute, Perelman School of Medicine, University of Pennsylvania Philadelphia PA 19104 USA; h Institute for Regenerative Medicine, Perelman School of Medicine, University of Pennsylvania Philadelphia PA 19104 USA

## Abstract

mRNA-based gene editing platforms have tremendous promise in the treatment of genetic diseases. However, for this potential to be realized *in vivo*, these nucleic acid cargos must be delivered safely and effectively to cells of interest. Ionizable lipid nanoparticles (LNPs), the most clinically advanced non-viral RNA delivery system, have been well-studied for the delivery of mRNA but have not been systematically optimized for the delivery of mRNA-based CRISPR-Cas9 platforms. In this study, we investigated the effect of microfluidic and lipid excipient parameters on LNP gene editing efficacy. Through *in vitro* screening in liver cells, we discovered distinct trends in delivery based on phospholipid, cholesterol, and lipid-PEG structure in LNP formulations. Combination of top-performing lipid excipients produced an LNP formulation that resulted in 3-fold greater gene editing *in vitro* and facilitated 3-fold greater reduction of a therapeutically-relevant protein *in vivo* relative to the unoptimized LNP formulation. Thus, systematic optimization of LNP formulation parameters revealed a novel LNP formulation that has strong potential for delivery of gene editors to the liver to treat metabolic disease.

## Abstract 2

Our work elucidates the importance of LNP formulation parameters, including microfluidic flow rate and organic excipient identity, for *in vivo* gene editing and presents an optimized delivery platform for the treatment of metabolic liver disease.

## Graphical abstract

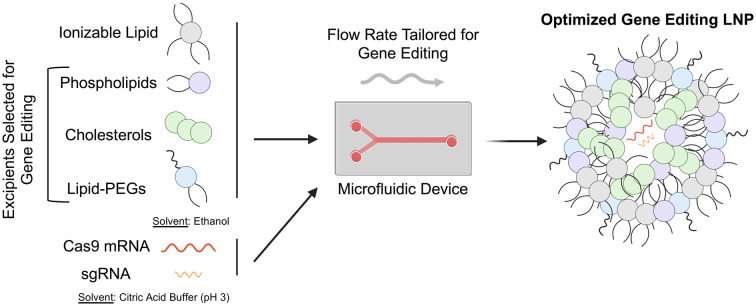

## Introduction

There are 10 000+ known monogenic diseases – disorders caused by mutations in a single gene – that together impact greater than 250 million people globally.^1^ These include pathologies of the central nervous system (*e.g.*, spinal muscular atrophy), lung (*e.g.*, cystic fibrosis), liver (*e.g.*, inborn errors of metabolism), and blood (*e.g.*, hemoglobinopathies). Few curative therapies exist for these diseases, and current clinical management focuses on symptom reduction.^2,3^

Genome editing involves the precise manipulation of DNA sequences to modulate resultant cellular phenotypes, offering a novel therapeutic strategy to cure monogenic diseases.^4^ CRISPR-Cas9, the most widely used genome editor, is an RNA-guided DNA-cutting enzyme complex that creates double-stranded DNA breaks (DSBs) at target positions in the DNA of eukaryotic cells.^4^ Repair of these breaks most commonly occurs *via* error-prone non-homologous end joining (NHEJ), which leads to small insertions and deletions (indels) at the break site that may interrupt gene function.^4^ Early clinical data suggest that NHEJ-mediated gene knockout can reduce the expression of disease-causing proteins or induce the expression of therapeutic proteins.^5,6^ Indeed, the FDA recently approved the first genome editing drugs that use CRISPR-Cas9 technology, Casgevy™ and Lyfgenia™, for the treatment of sickle cell disease and transfusion-dependent beta-thalassemia.^7^

To fully realize the therapeutic potential of genome editing, CRISPR-Cas9 must be delivered safely and effectively to target cells *in vivo*. Ionizable lipid nanoparticles (LNPs) are the most clinically advanced non-viral RNA delivery platform due to their excellent biocompatibility and efficacy in pre-clinical and clinical models.^8^ Conventionally, LNPs are composed of an RNA cargo that is microfluidically mixed with four organic components: i) an ionizable lipid that gains positive charge in acidic environments for RNA binding during nanoparticle formulation and subsequently releases RNA intracellularly at low endosomal pH, ii) a phospholipid to facilitate nanoparticle cellular uptake, iii) cholesterol for nanoparticle structural stability, and iv) a polyethylene glycol (PEG) coat to reduce opsonization and clearance of nanoparticles from the bloodstream.^3^ Many studies have investigated the optimal formulation parameters for LNP-mediated delivery of small interfering RNA (siRNA) and messenger RNA (mRNA).^9–11^ This has facilitated FDA approval of siRNA-LNP drugs (*e.g.*, Onpattro®) and mRNA-LNP vaccines (*e.g.*, Moderna and Pfizer SARS-CoV2 vaccines).^8^ However, few studies in the literature have systematically optimized LNPs for the delivery of mRNA-based CRISPR-Cas9 platforms.^12,13^

**Fig. 1:**
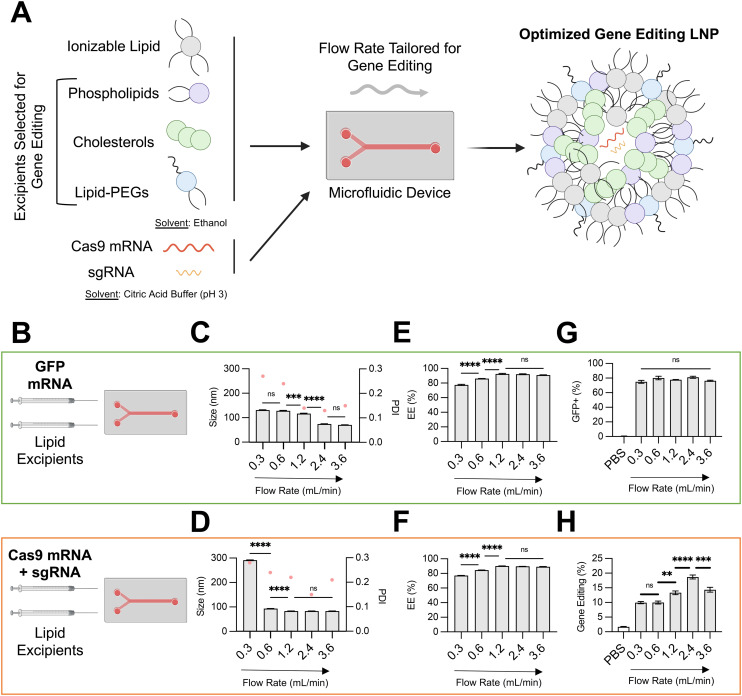
Optimization of LNP conditions for co-delivery of gene editing cargo. (A) Schematic describing the overall study design, where microfluidic and lipid excipient parameters were optimized to enhance LNP gene editing efficacy. (B) Depiction of the two nucleic cargos trialed and compared across microfluidic flow rates. (C) and (D) Size (left axis, bars) and PDI (right axis. pink dots) of LNPs produced at different total flow rates (0.3–3.6 mL min^−1^) encapsulating either GFP mRNA or SpCas9 mRNA and GFP sgRNA. (E) and (F) mRNA encapsulation efficiency of LNPs produced at different flow rates. (G) GFP transfection resulting from delivery of GFP mRNA to HepG2 cells *via* LNPs produced at different flow rates. (H) Gene editing resulting from delivery of Cas9 mRNA and GFP sgRNA to HepG2-GFP cells *via* LNPs produced at different flow rates. One-way ANOVA with *post hoc* Dunnett's test was used for statistical comparison (ns = non-significant, ** = *p* < 0.01, *** = *p* < 0.001, **** = *p* < 0.0001); all data reported as mean ± SEM (minimum *n* = 3).

Given the inherent chemical and structural differences between siRNA, mRNA, and mRNA-based CRISPR-Cas9 platforms in terms of size, stability, and charge density of the nucleic acids, we hypothesized that the optimal LNP formulation parameters to facilitate CRISPR-Cas9 gene editing would vary from those developed for siRNA or reporter mRNA delivery. Here, we studied both the microfluidic formulation parameters and organic excipient selection for LNPs co-encapsulating Cas9 mRNA and a single guide RNA (sgRNA) (Fig. 1A). Our *in vitro* optimization process yielded an LNP formulation that facilitated 3-fold greater therapeutic genome editing *in vivo* in the liver in comparison to an FDA-approved LNP formulation (MC3). Key features of the optimized LNP were the incorporation of new cholesterol and lipid-PEG analogs. Our work elucidates the importance of LNP formulation parameters for *in vivo* gene editing and presents an optimized delivery platform for the treatment of metabolic liver disease.

## Materials and methods

### Ionizable lipid synthesis

C14–494 ionizable lipids were synthesized as previously described.^14^ Briefly, 2-{2-[4-(2-{[2-(2-aminoethoxy)ethyl]amino}ethyl)piperazin-1-yl]ethoxy}ethan-1-amine (denoted as 494, Enamine, Kyiv, Ukraine) was reacted with excess 1,2 epoxytetradecane (denoted as C14, MilliporeSigma, Burlington, MA) in a 4 mL glass scintillation vial with a magnetic stir bar for 2 days at 80 °C. The product was transferred to a Rotovapor R-300 for solvent evaporation. For purification, lipid fractions were separated *via* a CombiFlash Nextgen 300+ chromatography system (Teledyn ISCO, Lincoln, NE). The fraction containing C14–494 ionizable lipid was identified *via* liquid chromatography-mass spectrometry (LC-MS). C14–494 ionizable lipid was suspended in ethanol prior to experimentation.

### mRNA production

GFP and SpCas9 mRNA were sourced from TriLink Biotechnologies (San Diego, CA) with CleanCap® modifications. GFP CRISPRevolution™ sgRNA was sourced from Synthego (Redwood City, CA) with a sequence of 5′– GGGCGAGGAGCUGUUCACCG – 3′. TTR sgRNA was sourced from Synthego (Redwood City, CA) with a sequence of 5′– UUACAGCCACGUCUACAGCA – 3′.

### LNP formulation and characterization

C14–494 ionizable lipid was combined in ethanol with cholesterol (MilliporeSigma), 1,2-dioleoyl-*sn-glycero*-3-phosphoethanolamine (DOPE, Avanti Polar Lipids, Alabaster, AL), and 1,2-dimyristoyl-*sn-glycero*-3-phosphoethanolamine-*N*-[methoxy(polyethylene glycol)-2000] (DMPE-PEG, Avanti Polar Lipids) to a total volume of 112.5 μL at a molar ratio of 35 : 46.5 : 16 : 2.5. A separate aqueous phase was prepared from 25 μg of GFP mRNA or 25 μg of a combination of SpCas9 mRNA and sgRNA (4 : 1 mass ratio) in 10 mM citrate buffer to a total volume of 337.5 μL. The ethanol and aqueous phases were chaotically mixed at flow rates specified in the main text *via* a soft lithography-patterned polydimethylsiloxane (PDMS) herringbone microfluidic device – fabricated as described in our previous study^15^ – to produce LNPs using a Pump33DDS dual drive syringe pump (Harvard Instruments, Holliston, MA). Specific channel dimensions for this device include a channel depth of 65 μm, an additional 25 μm depth for herringbones, a channel width of 200 μm, and a total mixing channel length of 31.5 mm. LNPs were dialyzed against 1× PBS in Slide-A-Lyzer G2 20 kDa dialysis cassettes (Thermo Fisher Scientific) for 2 h, sterilized using 0.22 or 0.45 μm filters, and stored at 4 °C. For MC3 LNPs, DLin-MC3-DMA ionizable lipid (Cayman Chemical, Ann Arbor, Michigan) was directly substituted for C14–494 ionizable lipid using the same excipients and formulation approach.

To produce phospholipid-substituted LNPs, DOPE was replaced during LNP formulation with either 1,2-distearoyl-*sn-glycero*-3-phosphocholine (DSPC, Avanti Polar Lipids), 1-stearoyl-2-oleoyl-*sn-glycero*-3-phosphocholine (SOPC, Avanti Polar Lipids) or 1,2-dioleoyl-*sn-glycero*-3-phosphocholine (DOPC, Avanti Polar Lipids). To produce cholesterol-substituted LNPs, cholesterol was replaced during LNP formulation with either 24-α-methyl-cholesterol (campesterol, Cayman Chemical), 24-α-ethyl-cholestanol (stigmastanol, Avanti Polar Lipids), or 24-α-ethyl-cholesterol (β-sitosterol, Avanti Polar Lipids). To produce PEG-substituted LNPs, DMPE-PEG was replaced during LNP formulation with either 1,2-distearoyl-*sn-glycero*-3-phosphoethanolamine-*N*-[methoxy(polyethylene glycol)-2000] (DSPE-PEG, Avanti Polar Lipids), 1,2-dimyristoyl-*rac-glycero*-3-methoxypolyethylene glycol-2000 (DMG-PEG, Avanti Polar Lipids), or methoxypolyethyleneglycoloxy(2000)-*N*,*N*-ditetradecylacetamide (DTA-PEG, Avanti Polar Lipids). To produce LNPs with multiple organic excipient substitutions, substitutions were made as indicated in the main text during LNP formulation.

DynaPro® Plate Reader III (Wyatt Technology, Santa Barbara, CA) was used to measure the *z*-average diameter and polydispersity index (PDI) of LNPs, while a Zetasizer Nano (Malvern Panalytical, Malvern, United Kingdom) was used to measure zeta potential of LNPs. Encapsulation efficiency and concentration of mRNA within each LNP formulation was measured after filtration using a Quant-iT-RiboGreen (Thermo Fisher Scientific) assay *via* manufacturer specifications. All LNP characterization data is reported as the mean of triplicate measurements. All materials were prepared and handled nuclease-free throughout synthesis, formulation, and characterization steps.

### *In vitro* assessment of gene editing LNPs

To test the *in vitro* efficacy of gene editing LNPs, HepG2-GFP cells were cultured in Dulbecco's modified Eagle's medium (DMEM) with l-glutamine (Gibco, Dublin, Ireland) supplemented with 10% volume/volume of fetal bovine serum (Gibco) and 1% volume/volume penicillin–streptomycin (Gibco). HepG2-GFP cells were seeded at a density of 20 000 cells per 100 μL. LNPs containing a total of 150 ng total RNA were used to treat cells, and cells were grown for a total of 5 days. At harvest, cells were isolated and resuspended in flow cytometry buffer (Ca^2+^/Mg^2+^ free PBS, 0.5% BSA, 0.5 mM EDTA). Samples were analyzed for fluorescence (GFP+) *via* flow cytometry (BD FACSAria™ Cell Sorter, Haryana, India). Viability was assessed in a duplicate plate of treated cells *via* Live/Dead™ Cytotoxicity Kit (Thermo Fisher Scientific). To test the *in vitro* efficacy of reporter mRNA LNPs, the same protocols were followed with HepG2-GFP cells replaced with HepG2 cells.

### *In vivo* assessment of gene editing LNPs

All animal use and protocols were approved by the Institutional Animal Care and Use Committee (IACUC) at the University of Pennsylvania (#806540) and followed guidelines of the National Institutes of Health's Guide for the Care and Use of Laboratory Animals. C57BL/6J mice (#000664) were purchased from The Jackson Laboratory (Bar Harbor, ME). Mice were housed in the Clinical Research Building at the University of Pennsylvania.

For *in vivo* gene editing experiments, adult female C57BL/6J mice were injected with LNPs encapsulating SpCas9 mRNA and TTR sgRNA at a dose of 1 mg kg^−1^ of total RNA *via* standard access of the lateral tail vein. After 5 days, tissues were harvested and genomic DNA was extracted using a DNeasy Blood and Tissue Kit according to manufacturer's instructions (Qiagen, Hilden, Germany). PCR amplification of the target amplicon was carried out using SuperFi II Hi-Fidelity DNA Polymerase (Thermo Fisher Scientific) with a universal annealing temperature of 60 °C and the following primer sequences: mTTR-exon2-F, 5′-CGGTTTACTCTGACCCATTTC-3′ and mTTR-exon2-R, 5′-GGGCTTTCTACAAGCTTACC-3′. Full-length Illumina sequencing adapters were then added to PCR products using a Nextera XT DNA Library Preparation Kit (Illumina, San Diego, CA). Pooled samples were sequenced using an Illumina MiSeq system. Alignment of fastq files to the target amplicon and quantification of editing frequency at the TTR locus was performed using CRISPResso2.

Mouse serum from experimental animals prior to LNP treatment and 5 days after LNP treatment was harvested and analyzed *via* ELISA for quantification of TTR protein (#OKIA00111, Aviva Systems Biology, San Diego, CA) per manufacturer specifications. Mouse serum was analyzed for AST and ALT activity *via* a Roche Cobas Chemistry Analyzer (Roche, Basel, Switzerland).

### Statistical analysis

All statistical analyses were carried out using GraphPad Prism 9 software. As described in figure captions, unpaired Student's *t* tests or one-way ANOVA with *post hoc* Dunnett's test were used to determine significance between experimental groups.

## Results and discussion

### Optimization of microfluidic total flow rate for LNP co-delivery of SpCas9 mRNA and sgRNA

Microfluidic methods based on stepwise ethanol dilution can generate LNPs with precisely defined physiochemical properties. Previously, we have used a microfluidic approach to generate LNPs encapsulating diverse nucleic acid cargos, including DNA, siRNA, reporter mRNA, and protein.^15–20^ In these studies, an ethanol-based solution of ionizable lipid and other organic excipients – phospholipid, cholesterol, lipid-PEG – and a buffered solution of nucleic acids were prepared, loaded into a glass syringes, and rapidly mixed. The mixing step was performed in a microfluidic device fabricated with polydimethylsiloxane (PDMS) and etched *via* soft lithography with channels designed to promote chaotic mixing. Syringe pumps were used to control the inlet flow rates, while the outlet of the microfluidic device was connected to a dialysis cassette for buffer exchange.

Using this microfluidic system, in this study, we investigated the effect of the total flow rate (TFR) of mixing on the physiochemical properties and *in vitro* efficacy of LNPs encapsulating either a GFP reporter mRNA (996 nucleotides) or both SpCas9 mRNA (4521 nucleotides) and GFP sgRNA (∼100 nucleotides) (Fig. 1B). We hypothesized that encapsulation of larger and more charge dense mRNA-based cargos would require more precise microfluidic control of LNP formulation. To isolate the effect of microfluidic TFR, the organic phase was prepared identically for both nucleic acid conditions, using a multi-tail piperazine-based ionizable lipid previously shown to facilitate mRNA delivery to the fetal liver (C14–494)^21^ and organic excipients (DOPE, cholesterol, DMPE-PEG) known to enhance intracellular mRNA delivery.^9^ The molar ratio of organic components (35 : 16 : 46.5 : 2.5) and N : P ratio between the ionizable lipid and mRNA (10 : 1) was also held constant for these experiments, given that these have been previously optimized for LNP-mediated mRNA delivery.^9^

The TFR in our microfluidic system was varied from 0.3 mL min^−1^ to 3.6 mL min^−1^, while the flow rate ratio (FRR) between the aqueous and organic phases were maintained at 3 : 1. For both LNPs encapsulating GFP mRNA and LNPs encapsulating SpCas9 mRNA and GFP sgRNA, an increase in TFR led to a corresponding decrease in average LNP diameter and PDI (Fig. 1C and D) and an increase in RNA encapsulation efficiency (EE%) (Fig. 1E and F). At slow TFR nearing the mixing rate of physical methods (*e.g.*, pipette mixing), we observed a significantly larger size for LNP encapsulating gene editing cargo (∼300 nm) in comparison to reporter mRNA, likely due to electrostatic interactions between larger and more negative nucleic cargo with positively charged ionizable lipid. These trends align with those previously described during formulation of lipid-based nanoparticles.^22,23^ To investigate the *in vitro* transfection efficacy of LNPs prepared at different TFR, either HepG2 cells (immortalized human hepatoma cell line) or HepG2-GFP cells (HepG2 cell line constitutively expressing GFP) were treated with LNPs at a dose of 150 ng total RNA per 20 000 cells. Transfection for LNPs encapsulating GFP mRNA was quantified *via* flow cytometry by resultant GFP expression in HepG2 cells, while transfection for LNPs encapsulating SpCas9 mRNA and GFP sgRNA was quantified by knockout of GFP expression in HepG2-GFP cells.

Variation of TFR to produce LNPs encapsulating GFP mRNA did not alter *in vitro* transfection efficacy in HepG2 cells (Fig. 1G). In contrast, an increase in TFR from 0.3 mL min^−1^ to 2.4 mL min^−1^ resulted in gene editing LNPs that mediated nearly 2-fold greater editing at the GFP locus in HepG2-GFP cells (Fig. 1H). For gene editing LNPs, low TFR may lead to early precipitation of lipid components within the microfluidic channel, resulting in sub-optimal self-assembly into LNPs.^24^ Indeed, gene editing LNPs produced at a TFR of 0.3 mL min^−1^ were large (300 nm), heterogenous (PDI > 0.2), and poorly encapsulated their RNA cargo (<80%). Notably, at the highest TFR that was tested (3.6 mL min^−1^), LNPs had slightly decreased *in vitro* gene editing efficacy. While LNPs produced at a TFR of 3.6 mL min^−1^ had macro-scale physiochemical properties (*e.g.*, size, EE%) akin to those produced at 2.4 mL min^−1^, higher TFR may lead to increased shear stress during LNP formulation (evidenced by slightly higher PDI),^25^ leading to modulation of internal lipid bilayer structure and RNA packing and ultimately negatively impacting delivery of gene editing cargos due to incomplete RNA encapsulation and lower stability.^26^ Although further study of the internal structure of these LNPs prepared at different TFR is necessary (*e.g.*, transmission electron microscopy, small-angle X-ray scattering), based on these data, an optimal TFR of 2.4 mL min^−1^ was maintained for downstream formulation of LNPs with varying organic excipient identities.

### Impact of phospholipid structure on LNP-mediated gene editing

The structural and biological properties of LNPs are not solely ascribed to a single lipid excipient component. Rather, each lipid excipient component plays a fundamental role in LNP bioactivity.^3^ The ionizable lipid typically possesses a tertiary amine that is protonated in pH conditions below the acid-dissociation constant (p*K*_a_) of the lipid and deprotonated in neutral conditions.^8^ Ionizable lipid pH sensitivity enables nucleic acid encapsulation during formulation and nucleic acid release *via* endosomal disruption. In this study, we chose C14–494 ionizable lipid for all LNP formulations given strong *in vitro* and *in vivo* evidence of mRNA delivery efficacy to the fetal liver.^21^

Phospholipids play several critical roles in LNP delivery, including enhancing endosomal escape, aiding in solubilization of RNAs inside aqueous pockets, and even driving organ tropism *in vivo*.^27^ Computational and experimental studies have suggested that phospholipids with phosphatidylcholine (PC) head groups provide stability to the bilayer membrane, while those with phosphatidylethanolamine (PE) head groups introduce membrane curvature, increase tension, and, in turn, endosomal disruption.^28^ The relative saturation of phospholipid tails can also modulate the fluidity of the lipid bilayer and promote either the formation of a less stable hexagonal phase (unsaturated tails) or a more stable lamellar phase (saturated tails).^29^ However, the selection of phospholipids in LNPs co-encapsulating SpCas9 mRNA and sgRNA has not been well-studied.

**Fig. 2:**
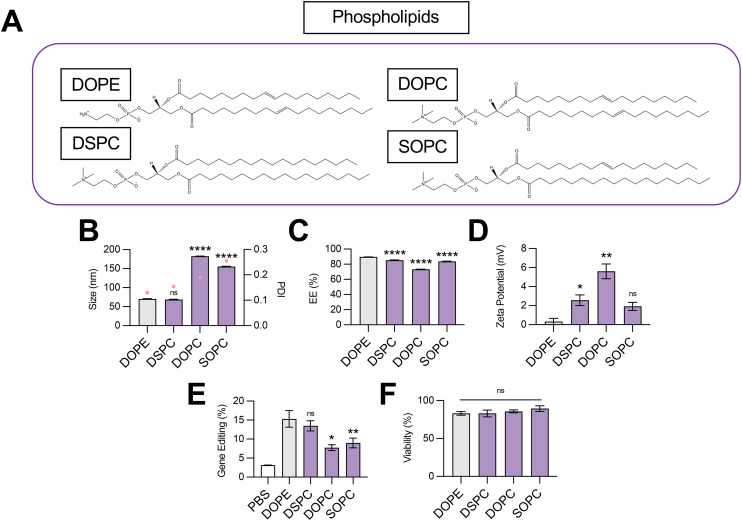
Optimization of phospholipid structure for co-delivery of gene editing cargo. (A) Structures of phospholipids incorporated into LNPs. (B) Size (left axis, bars) and PDI (right axis, pink dots), (C) RNA encapsulation efficiency (EE%), and (D) zeta potential of LNPs produced with different phospholipids to encapsulate SpCas9 mRNA and GFP sgRNA. (E) Gene editing resulting from delivery of Cas9 mRNA and GFP sgRNA to HepG2-GFP cells *via* LNPs produced with different phospholipids. (F) Viability of cells treated with LNPs produced with different phospholipids. One-way ANOVA with *post hoc* Dunnett's test was used for statistical comparison relative to DOPE LNPs (ns = non-significant, * = *p* < 0.05, ** = *p* < 0.01, **** = *p* < 0.0001); all data reported as mean ± SEM (minimum *n* = 5).

To probe the effect of phospholipid structure on the gene editing efficacy of LNPs, we formulated C14–494 LNPs to encapsulate SpCas9 mRNA and GFP sgRNA with DSPC, DOPC, or SOPC substituted for DOPE (Fig. 2A). In comparison to DOPE, a PE phospholipid with unsaturated lipid tails, DSPC is a PC phospholipid with saturated lipid tails, while DOPC and SOPC are PC phospholipids with unsaturated lipid tails. DOPE, DSPC, DOPC, and SOPC LNPs were characterized for size (Fig. 2B), EE% (Fig. 2C), and zeta potential (Fig. 2D). DOPE LNPs had an average diameter of 70 nm, which was similar to the size of DSPC LNPs (68 nm) but significantly smaller than DOPC LNPs (182 nm) and SOPC LNPs (155 nm) (Fig. 2B). Although DOPE LNPs boasted the highest EE% (89%), all LNP formulations had greater than 80% encapsulation efficiency (Fig. 2C). DOPE LNPs had a nearly neutral surface zeta potential (+0.33 mV), while DSPC, DOPC, and SOPC LNPs all had positive zeta potential values (Fig. 2D). In HepG2-GFP cells, DOPE LNPs and DSPC LNPs both facilitated gene editing at similar rates, resulting in GFP knockout in 15% of cells (Fig. 2E). In contrast, DOPC and SOPC LNPs mediated gene editing in only ∼8% of cells. All LNP formulations resulted in greater than 80% cell viability (Fig. 2F).

Our data convey that inclusion of DOPE and DSPC in the organic phase produces LNPs with similar physiochemical properties and gene editing efficacy *in vitro*. The smaller size of DOPE LNPs and DSPC LNPs likely allows for enhanced cellular uptake,^30^ leading to greater intracellular delivery of gene editing cargos. LNPs produced with PC containing phospholipids mediated higher levels of gene editing as saturation of lipid tails increased (DOPC → SOPC → DSPC), corresponding to trends observed in zeta potential (Fig. 2E). With more saturated hydrocarbon chains, the phospholipid assumes a more cylindrical shape,^31^ tending toward the formation of a more stable bilayer phase, which may be necessary to better encapsulate large mRNA cargos. In contrast, when comparing the impact of saturated phospholipids with distinct head groups on LNP gene editing efficacy, LNPs formulated with PE head group phospholipid (DOPE) outperformed those formulated with PC head group phospholipid (DOPC) by 2-fold (Fig. 2E), possibly due to promotion of a non-bilayer inverted hexagonal phase, which facilitates improved endosomal membrane fusion of phospholipids.^32^ Thus, there appears to be a balance, and likely a complex interplay, between lipid bilayer stabilizing and destabilizing properties conveyed by phospholipids for maximal delivery of gene editing cargo. More broadly, these data imply that either partially or fully saturated phospholipids with a PE head group such as 1-stearoyl-2-oleoyl-*sn-glycero*-3-phosphoethanolamine (SOPE) or 1,2-distearoyl-*sn-glycero*-3-phosphoethanolamine (DSPE) may further optimize LNPs for gene editing applications.

### Impact of cholesterol structure on LNP-mediated gene editing

Cholesterol plays several important roles in LNPs. The tetracyclic core of cholesterol confers a planar structure that allows for intercalation into bilayer structures,^33^ which decreases membrane permeability and affords the bilayer a greater resistance to destabilization by serum components.^31^ The inclusion of cholesterol in LNPs has also been shown to be fundamental in nucleic acid delivery, potentially by promoting endosomal membrane fusion and interaction with intracellular trafficking proteins that direct endosomal escape and nanoparticle release into the cytosol.^33–35^ Recently, structure–activity analysis of cholesterol analogs revealed that incorporation of C-24 alkyl phytosterols into LNPs in the place of cholesterol enhanced mRNA transfection.^36^ Here, we assessed the effect of cholesterol substitution with three analogs on LNP gene editing efficacy.

**Fig. 3:**
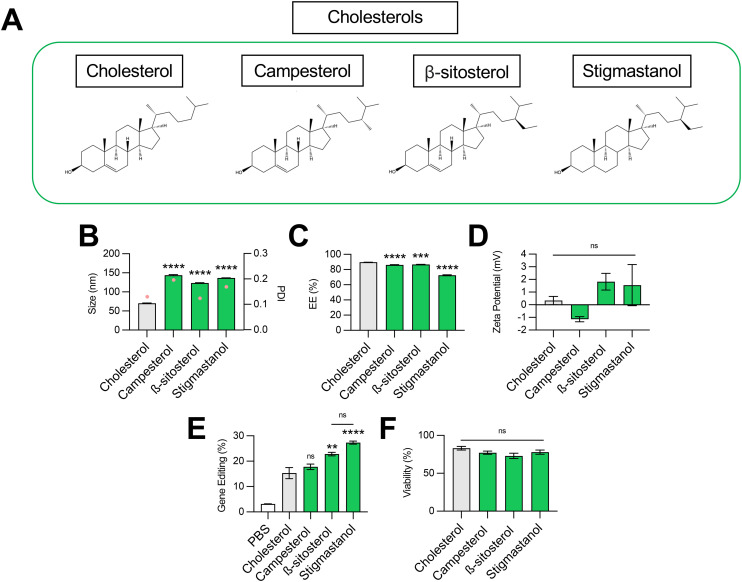
Optimization of cholesterol structure for co-delivery of gene editing cargo. (A) Structures of cholesterols incorporated into LNPs. (B) Size (left axis, bars) and PDI (right axis, pink dots), (C) RNA encapsulation efficiency (EE%), and (D) zeta potential of LNPs produced with different cholesterols to encapsulate SpCas9 mRNA and GFP sgRNA. (E) Delivery of Cas9 mRNA and GFP sgRNA to HepG2-GFP cells *via* LNPs produced with different cholesterols. (F) Viability of cells treated with LNPs produced with different cholesterols. One-way ANOVA with *post hoc* Dunnett's test was used for statistical comparison relative to cholesterol LNPs (ns = non-significant, ** = *p* < 0.01, *** = *p* < 0.001, **** = *p* < 0.0001); all data reported as mean ± SEM (minimum *n* = 5).

To this end, we formulated C14–494 LNPs to encapsulate SpCas9 mRNA and GFP sgRNA with campesterol, β-sitosterol, and stigmastanol (Fig. 3A) substituted for cholesterol. In comparison to cholesterol, campesterol and β-sitosterol possess alkyl substitutions in the tail region, while stigmastanol has both an alkyl-substituted tail and reduction of a double bond within a sterol ring in the body region. Cholesterol, campesterol, β-sitosterol, and stigmastanol LNPs were characterized for size (Fig. 3B), EE% (Fig. 3C), and zeta potential (Fig. 3D). All three cholesterol analogs doubled the size of LNPs (∼140 nm) from the original cholesterol formulation (70 nm) (Fig. 3B) with a corresponding reduction in encapsulation efficiency (Fig. 3C). Substitution with cholesterol analogs had no effect on the surface zeta potential of LNPs, as all formulations remained nearly neutral (Fig. 3D). In HepG2-GFP cells, β-sitosterol LNPs and stigmastanol LNPs enhanced gene editing efficacy by nearly 2-fold relative to the original cholesterol formulation (Fig. 3E). In contrast, campesterol LNPs mediated similar levels of knockout to cholesterol LNPs. There was no impact of cholesterol substitution on cell viability (Fig. 3F).

Previous studies have shown that the C-24 alkyl group in cholesterol imparts crystal defects in the ordering of the lipid bilayer and that the frequency of defects is directly proportional to the length of the alkyl side chain.^37^ LNPs produced with C-24 alkyl analogs may have a more faceted surface geometry than the relatively uniform curvature of cholesterol LNPs, facilitating cellular uptake and potentially biasing subcellular trafficking toward pathways that promote endosomal escape.^36^ Our results demonstrate that gene editing was enhanced with the introduction of the longer C-24 ethyl group (β-sitosterol, stigmastanol) relative to a C-24 alkyl group (campesterol) or the native, unsubstituted tail (cholesterol) (Fig. 3E). As has been shown previously for reporter mRNA delivery, the gene editing efficacy of C-24 ethyl cholesterol analogs did not vary with the presence of a Δ − 5 double bond (β-sitosterol *vs.* stigmastanol),^36^ demonstrating that the flexibility endowed by saturation of this double bond does not impact LNP gene editing efficacy. Together, our results corroborate that cholesterol analogs can enhance mRNA transfection and, more specifically, improve the delivery of mRNA-based gene editing cargos.

### Impact of lipid-PEG structure on LNP-mediated gene editing

The incorporation of a hydrophilic lipid-PEG into LNPs provides a steric barrier that drives lipid bilayer self-assembly during formulation.^38^ Lipid-PEGs are widely used to enhance *in vivo* pharmacokinetic properties of LNPs, including serum stability, circulation half-life, and evasion of the mononuclear phagocyte system.^39^ However, PEGylation has also been shown to negatively impact LNP nucleic acid delivery, since the addition of lipid-PEG introduces significant steric hindrance and subsequently hinders cellular uptake and endosomal escape.^40^ Given these competing effects, we hypothesized that the choice of lipid-PEG would impact the gene editing efficacy of LNPs.

**Fig. 4:**
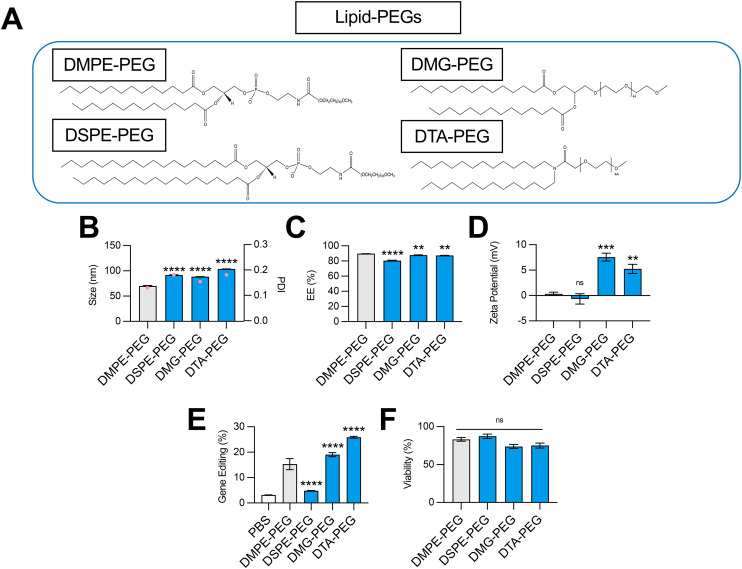
Optimization of lipid-PEG structure for co-delivery of gene editing cargo. (A) Structures of lipid-PEGs incorporated into LNPs. (B) Size (left axis, bars) and PDI (right axis, pink dots), (C) RNA encapsulation efficiency (EE%), and (D) zeta potential of LNPs produced with different lipid-PEGs to encapsulate SpCas9 mRNA and GFP sgRNA. (E) Delivery of Cas9 mRNA and GFP sgRNA to HepG2-GFP cells *via* LNPs produced with different lipid-PEGs. (F) Viability of cells treated with LNPs produced with different lipid-PEGs. One-way ANOVA with *post hoc* Dunnett's test was used for statistical comparison relative to DMPE-PEG LNPs (ns = non-significant, ** = *p* < 0.01, *** = *p* < 0.001, **** = *p* < 0.0001); all data reported as mean ± SEM (minimum *n* = 5).

To test this, we formulated C14–494 LNPs to encapsulate SpCas9 mRNA and GFP sgRNA with DSPE-PEG, DMG-PEG, or DTA-PEG (Fig. 4A) substituted for DMPE-PEG. In comparison to DMPE-PEG (C14), DSPE-PEG (C18) possesses an additional four methylene groups in its acyl tails. DMG-PEG and DTA-PEG utilize a glycerol linker or an amide linker, respectively, instead of the *glycero*-3-phosphoethanolamine linkage used in DMPE-PEG between the lipophilic tails and the PEG chain. Notably, DMG-PEG is the lipid-PEG used in the formulation of the Pfizer SARS-CoV-2 vaccine, while DTA-PEG is the lipid-PEG used in the formulation of the Moderna SARS-CoV-2 vaccine.^8^ All lipid-PEGs had an average polymer molecular weight of 2000 (PEG-2000). DMPE-PEG, DSPE-PEG, DMG-PEG, and DTA-PEG LNPs were characterized for size (Fig. 4B), EE% (Fig. 4C), and zeta potential (Fig. 4D). Substitution of DMPE-PEG with DSPE-PEG, DMG-PEG, or DTA-PEG PEG resulted in an increase in LNP size from 70 nm to 90–100 nm (Fig. 4B), while encapsulation efficiency remained above 80% for all LNP formulations (Fig. 4C). Like DMPE-PEG LNPs, DSPE-PEG LNPs had a neutral surface zeta potential, while DMG-PEG and DTA-PEG PEG LNPs had a positive surface zeta potential between 5–7 mV (Fig. 4D). In HepG2-GFP cells, DSPE-PEG LNPs mediated 3-fold less GFP knockout relative to DMPE-PEG LNPs, while DMG-PEG LNPs and DTA-PEG LNPs significantly enhanced gene editing efficacy (Fig. 4E). None of these LNPs were cytotoxic (Fig. 4F).

Given their linker chemistries, DMPE-PEG and DSPE-PEG can be classified as anionic lipid-PEGs, while DMG-PEG and DTA-PEG can be classified as neutral lipid-PEGs. In line with their charge properties, LNPs coated with DMG-PEG and DTA-PEG had more positive surface zeta potentials than DMPE-PEG and DSPE-PEG LNPs (Fig. 4D). Interestingly, LNPs produced with neutral lipid-PEGs also facilitated higher levels of gene editing (Fig. 4E). This result may be attributed to less favorable charge interactions between anionic lipid-PEGs and large, highly anionic Cas9 mRNA and sgRNA cargo and/or electrostatic interactions between positive surface charge LNPs and negatively charged cell membranes that promote greater cellular uptake. Relative to DMPE-PEG LNPs, DSPE-PEG LNPs demonstrated poor gene editing efficacy (Fig. 4E). Although DSPE-PEG is less easily shed *in vivo*, allowing for extension of circulation time,^41,42^ longer lipid-PEG tails have been shown to decrease cellular uptake,^43^ supporting the observed decrease in LNP transfection efficacy. Thus, for hepatic gene editing applications, the selection of a shorter lipid-PEG (DMPE-PEG, DMG-PEG, DTA-PEG) in LNP formulations maximizes both cellular uptake and liver targeting due to rapid dissociation and replacement with an ApoE rich protein corona to facilitate effective liver targeting.^36^ However, for extrahepatic gene editing applications, selection of a lipid-PEG may need to be more nuanced, considering competing contributions of this excipient to both *in vivo* biodistribution and transfection.

### Effect of excipient combinations on LNP-mediated gene editing *in vitro* and *in vivo*

**Fig. 5:**
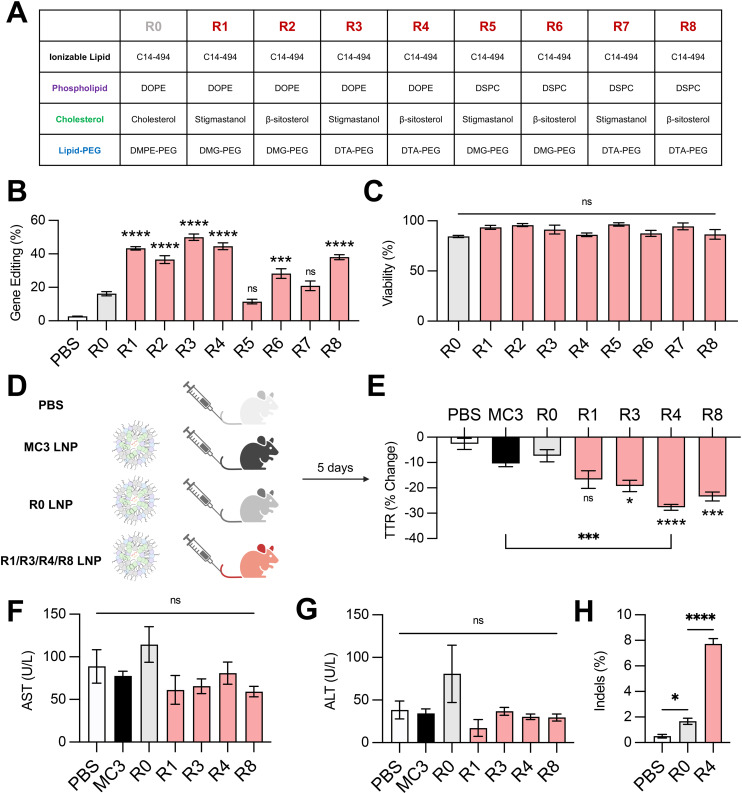
Delivery of gene editing cargo *in vitro* and *in vivo* with excipient-optimized LNPs. (A) Table depicting formulations for excipient-optimized LNPs using the top-performing phospholipids (DOPE, DSPC), cholesterols (stigmastanol, β-sitosterol), and lipid-PEGs (DMG-PEG, DTA-PEG). Formulations are labeled R1–R8. Base formulation is represented as R0. (B) Delivery of Cas9 mRNA and GFP sgRNA to HepG2-GFP cells *via* LNPs produced with different combinations of excipients. (C) Viability of cells treated with LNPs produced with different combinations of excipients. (D) Experimental groups for *in vivo* assessment of LNP gene editing efficacy. PBS was used as a negative control. MC3 LNPs are an FDA-approved LNP formulation. R1, R3, R4, and R8 LNPs were the top-performers from the *in vitro* screen and were compared against R0 LNPs. All LNPs were formulated to encapsulate SpCas9 mRNA and TTR sgRNA. (E) C57BL/6 mice were treated with LNPs at a dose of 1 mg kg^−1^ and sacrificed after 5 days. Serum was collected before and after LNP treatment and analyzed for TTR protein *via* ELISA. (F) AST and (G) ALT levels in the serum of mice treated with LNPs relative to PBS-injected controls. (H) Next-generation sequencing analysis for insertions and deletions (indels) at the expected locus for TTR gene editing in liver genomic DNA of PBS-, R0 LNP-, or R4 LNP-treated animals. One-way ANOVA with *post hoc* Dunnett's test was used for statistical comparison relative to R0 LNPs unless otherwise denoted (ns = non-significant, * = *p* < 0.05, *** = *p* < 0.001, **** = *p* < 0.0001); all data reported as mean ± SEM (minimum *n* = 5).

Next, we investigated the effect of combining the top-performing lipid excipients in distinct LNP formulations. We formulated all possible combinations (Fig. 5A) of C14–494 LNPs encapsulating SpCas9 mRNA and GFP sgRNA using the best two phospholipids (DOPE and DSPC), cholesterol analogs (stigmastanol and β-sitosterol), and lipid-PEGs (DMG-PEG and DTA-PEG). The eight formulations (R1–R8) were screened against the base C14–494 LNP formulation (R0) in HepG2-GFP cells. Six out of eight LNPs (R1, R2, R3, R4, R6, R8) enhanced GFP knockout in HepG2-GFP cells relative to R0 LNPs (Fig. 5B). The top four LNPs (R1, R3, R4, R8) resulted in greater than two-fold gene editing at the GFP locus *in vitro* (Fig. 5B) without cellular toxicity (Fig. 5C). Notably, R1, R3, R4, and R8 LNPs all achieved higher levels of gene editing than any LNP formulation with a single lipid excipient changed (Fig. 2E, 3E and 4E).

To test the *in vivo* efficacy of the lead LNP formulations, we chose the *TTR* gene as a representative target. The *TTR* gene is currently being investigated in clinical trials for patients with hereditary transthyretin amyloidosis.^5^ Knockout of the *TTR* gene leads to decreased levels of misfolded TTR protein and thereby limits the buildup of pathogenic amyloid plaques. We re-formulated R1, R3, R4, and R8 LNPs to encapsulate SpCas9 mRNA and TTR sgRNA, alongside the base LNP formulation (R0) and an FDA-approved LNP formulation (MC3) as controls. All LNPs were administered *via* tail vein injection in 8 week-old C57BL/6 mice at a dose of 1 mg kg^−1^ total RNA (Fig. 5D). TTR protein levels were assessed before and after treatment *via* serum ELISA. Three out of four excipient-optimized LNPs (R3, R4, and R8) resulted in significantly greater reduction in serum TTR protein in comparison to the base R0 LNP formulation (Fig. 5E). Treatment with R4 LNPs also resulted in significantly greater TTR knockdown than the clinically-approved MC3 LNP formulation (Fig. 5E). None of the LNPs that were tested *in vivo* led to elevations in hepatic enzymes, demonstrating safety of these delivery carriers (Fig. 5F and G). The livers of mice treated with PBS, R0 LNPs, or R4 LNPs were subsequently harvested and digested to isolate genomic DNA. Next-generation sequencing analysis of these samples at the expected locus of genome editing within the TTR gene revealed that optimized R4 LNPs outperformed unoptimized R0 LNPs by nearly 4-fold (Fig. 5H). Thus, our top-performing LNP formulation – optimized systematically for lipid excipients to enhance delivery of gene editing cargo – demonstrates strong potential for mRNA-based gene editing therapies in the liver.

## Conclusions

In this study, we assessed the effect of microfluidic and lipid excipient parameters on LNP co-delivery of mRNA-based gene editing cargos. Although LNPs encapsulating reporter mRNA were not sensitive to the microfluidic mixing conditions tested, an optimal total flow rate was identified for LNPs encapsulating SpCas9 mRNA and sgRNA. Future work will involve probing the effect of the microfluidic flow rate ratio between the lipid and mRNA phases and channel architecture on LNP gene editing efficacy, the influence of excipient identity on organ biodistribution, and the translation of these results to a range of ionizable lipids. Testing each lipid excipient independently, we also discovered trends in LNP gene editing efficacy with the incorporation of distinct phospholipids, cholesterol analogs, and lipid-PEGs, although the exact mechanism by which these lipid excipients enhance gene editing warrants further study. Of note, a subset of C-24 alkyl phytosterols and neutral lipid-PEGs vastly improved LNP-mediated gene editing *in vitro* in liver cells. Combining optimal excipient structures resulted in a lead LNP formulation (R4 LNPs) that improved liver gene editing and knockout of therapeutically-relevant protein *in vivo* relative to the original LNP formulation and an FDA-approved LNP formulation, demonstrating its potential for the delivery of gene editing therapeutics in the liver. Using design-of-experiments to determine optimal lipid molar ratios for LNP gene editing, adding a fifth lipid component to change LNP organ tropism, or conjugating a peptide or antibody fragment to improve LNP cellular uptake may produce further optimized LNPs tailored for a range of gene editing applications, including application *in utero* for the treatment of congenital liver disease.

## Data availability

All study data are included in the main text of this article.

## Author contributions

R. P., W. H. P., and M. J. M. designed research; R. P., E. L. H., K. L. S., A. M. M., R. M., D. K., S. T., H. Y., H. C. S., and K. M. performed research; R. P., W. H. P., and M. J. M. analyzed data; R. P., E. L. H., W. H. P., and M. J. M. wrote the manuscript.

## Conflicts of interest

R. P. and M. J. M. are inventors on a patent filed by the Trustees of the University of Pennyslvania based on technology detailed in this manuscript.
